# Evaluating the safety and effectiveness of α-blockers versus mirabegron for medical expulsive therapy in ureteral calculi: A Systematic review and meta-analysis

**DOI:** 10.1371/journal.pone.0315328

**Published:** 2024-12-27

**Authors:** Huilei Yan, Xiaoni Li, Xiaobo Zheng, Yuanshan Cui, Jing Huang, Yan Cheng

**Affiliations:** 1 Department of Urology, Liaocheng People’s Hospital, Liaocheng, China; 2 Department of Urology, Yantai Yuhuangding Hospital Affiliated to Medical College of Qingdao University, Yantai, China; 3 Qingdao Central Hospital, University of Health and Rehabilitation Sciences (Qingdao Central Medical Group), Qingdao, China; 4 Department of Nephrology, Yantai Yuhuangding Hospital Affiliated to Medical College of Qingdao University, Yantai, China; Stanford University School of Medicine, UNITED STATES OF AMERICA

## Abstract

**Introduction and aim:**

The main categories of drugs employed for medical expulsive therapy in patients with ureteral calculi (UC) are alpha-blockers (α-B) and beta-adrenoceptor agonists. This meta-analysis evaluated the safety and effectiveness of α-B versus mirabegron (MIR) in treating UC.

**Methods:**

From January 1980 to October 2024, we extensively searched the Pubmed, Web of science, Cochrane and EMBASE databases to identify randomized controlled trials (RCTs) that compared the effectiveness of α-B and MIR in managing UC. Furthermore, a systematic review and meta-analysis were carried out.

**Results:**

The meta-analysis included six publications with 592 patients, comparing α-B with MIR. The stone expulsion rate (SER) was found to be significantly greater in the α-B group than in the MIR group, as indicated by an odds ratio (OR) of 1.51 (95% confidence interval [CI]: 1.05 to 2.16, P = 0.03) in the meta-analysis. However, no significant differences were found between the α-B group and the MIR group for stone expulsion time (SET) (mean difference [MD]: 1.20; 95% CI, -2.71 to 5.10; P = 0.55), pain episodes (PE) (MD: 0.36; 95% CI, -0.04 to 0.76; P = 0.07), or analgesic requirements (MD: 0.79; 95% CI, -0.37 to 1.94; P = 0.18). The α-B group exhibited a significantly higher incidence of adverse events compared to the MIR group for orthostatic hypotension (OR 12.16, 95% CI 3.36 to 43.95, P = 0.0001), headache (OR 3.46, 95% CI 1.41 to 8.49, P = 0.007), and retrograde ejaculation (OR 16.30, 95% CI 5.87 to 45.31, P < 0.00001). While in the dizziness (OR 1.65, 95% CI 0.67 to 4.09, p = 0.28), it made no difference.

**Conclusions:**

Our meta-analysis identified a substantial enhancement in the SER among patients with UC who received α-B therapy instead of those who were administered MIR therapy. Nonetheless, α-B therapy was connected to an increased risk of adverse events.

**Systematic review registration:**

PROSPERO, ID CRD42024595934.

## Introduction

Urolithiasis, a prevalent global health issue, is of increasing significance due to its rising incidence and prevalence [1, 2]. The rise in obesity, diabetes, and metabolic syndrome is primarily attributed as the reason [3]. The clinical context determines the management of ureteral calculi (UC), which encompasses medical expulsive therapy (MET), observation, shockwave lithotripsy (SWL), percutaneous nephrolithotomy (PNL), ureteroscopy, as well as open and laparoscopic stone surgery [4]. MET is often utilized to hasten the passage of stones through the ureter, thereby averting ureteral obstruction, easing ureteral colic, and eliminating the need for surgical or more invasive interventions [5]. Various drugs, such as alpha-blockers (α-B), calcium channel blockers, phosphodiesterase (PDE) inhibitors, nonsteroidal anti-inflammatory drugs, β-adrenoceptor agonists, serotonergic drugs, corticosteroids, and combinations of these are utilized in MET for the clinical management of UC [6, 7].

The first high-quality RCT to evaluate the efficacy of α-B for distal UC measuring 7 mm or less was carried out by Hermans in 2009. According to the authors, tamsulosin’s sole advantage was its capacity to reduce the requirement for analgesics until the stone passed [8]. α-B is effective in treating UC, as indicated by the most recent systematic review and meta-analysis [9–11]. Furthermore, the American Urological Association and the European Association of Urology strongly advocate the provision of α-B to facilitate the passage of UC for patients [4, 12]. The hypothesized mechanism of action for α-B involves the inhibition of smooth muscle contraction in the ureter, leading to relaxation of the ureteral smooth muscle and a reduction in the intensity and frequency of peristalsis [13].

Mirabegron (MIR), a selective beta-adrenergic agonist, is employed in the management of over active bladder (OAB) [14]. The urothelium and smooth muscle of the human ureter express beta-adrenergic receptors (AR), and their activation leads to the relaxation of the human ureter [15]. Thus, many studies have demonstrated MIR’s efficacy in treating UC [16–18]. A significant improvement in the stone expulsion rate (SER) and decreased pain episodes (PE) were associated with MIR, as revealed by a new meta-analysis [19].

Furthermore, a systematic meta-analysis has not been conducted to evaluate the safety and effectiveness of α-B versus MIR for the management of UC. As a result, a meta-analysis was performed in this study to compare the effects of α-B and MIR in patients with UC.

## Materials and methods

This systematic review and meta-analysis were conducted following the PRISMA guidelines (Preferred Reporting Items for Systematic Reviews and Meta-Analyses).

### Studies

This meta-analysis aimed to evaluate the safety and effectiveness of α-B versus MIR for the management of UC. All of the RCTs we identified included participants who had a single and unilateral ureter stones in size between 4 and 10 mm. The planned MET interventions were α-B versus MIR. The α-B (Silodosin 8 mg or Tamsulosin 0.4mg) and MIR (Mirabegron 50 mg) were administered once daily. The MET was continued until stone passage or maximally for longest follow-up time ranged from 3weeks to 30days. The patients were advised to take a tablet of analgesic medication only during pain attacks. The patients who failed to pass stones at the end of the experiment discontinued MET and began surgical intervention. Besides, the MET was abort in some RCTs, if there was an infection, obstruction, resistance or challenge to manage pain, or worsening of renal function. Stone expulsion rate (SER) and stone expulsion time (SET) were considered as the primary outcomes. We also considered PE, analgesic requirements, and adverse events as the secondary outcome measures.

### Inclusion and exclusion criteria

The RCTs were required to meet specific criteria for inclusion: (1) they had to investigate the efficacy and safety of α-B compared to MIR for treating UC; (2) they needed to provide sufficient data for analysis, including SER, stone expulsion time (SET), PE, analgesic requirements, and adverse events; (3) access to the full text of the study had to be available. The exclusion criteria comprised studies that met the following conditions: (1) absence of available data, (2) inclusion of duplicate data, (3) presence of subsequent updated publications, and (4) lack of merged analysis data.

### Search strategy

Studies published between January 1980 and October 2024 were systematically searched for across the databases of Pubmed, Web of science, Cochrane and EMBASE without language or publication status restrictions. After obtaining the studies, we scrutinized their reference lists to pinpoint any RCTs that evaluated the effectiveness and safety of α-B versus MIR for MET in patients with UC. In the study, the following keywords were utilized: randomized controlled trials, alpha-blockers, ureteral calculi, urolithiasis, ureteral stone, silodosin, tamsulosin, alfuzosin, terazosin, doxazosin, mirabegron, and MET. The search also encompassed abbreviations, including α-B and RCTs.

### Trial selection

**Fig 1:**
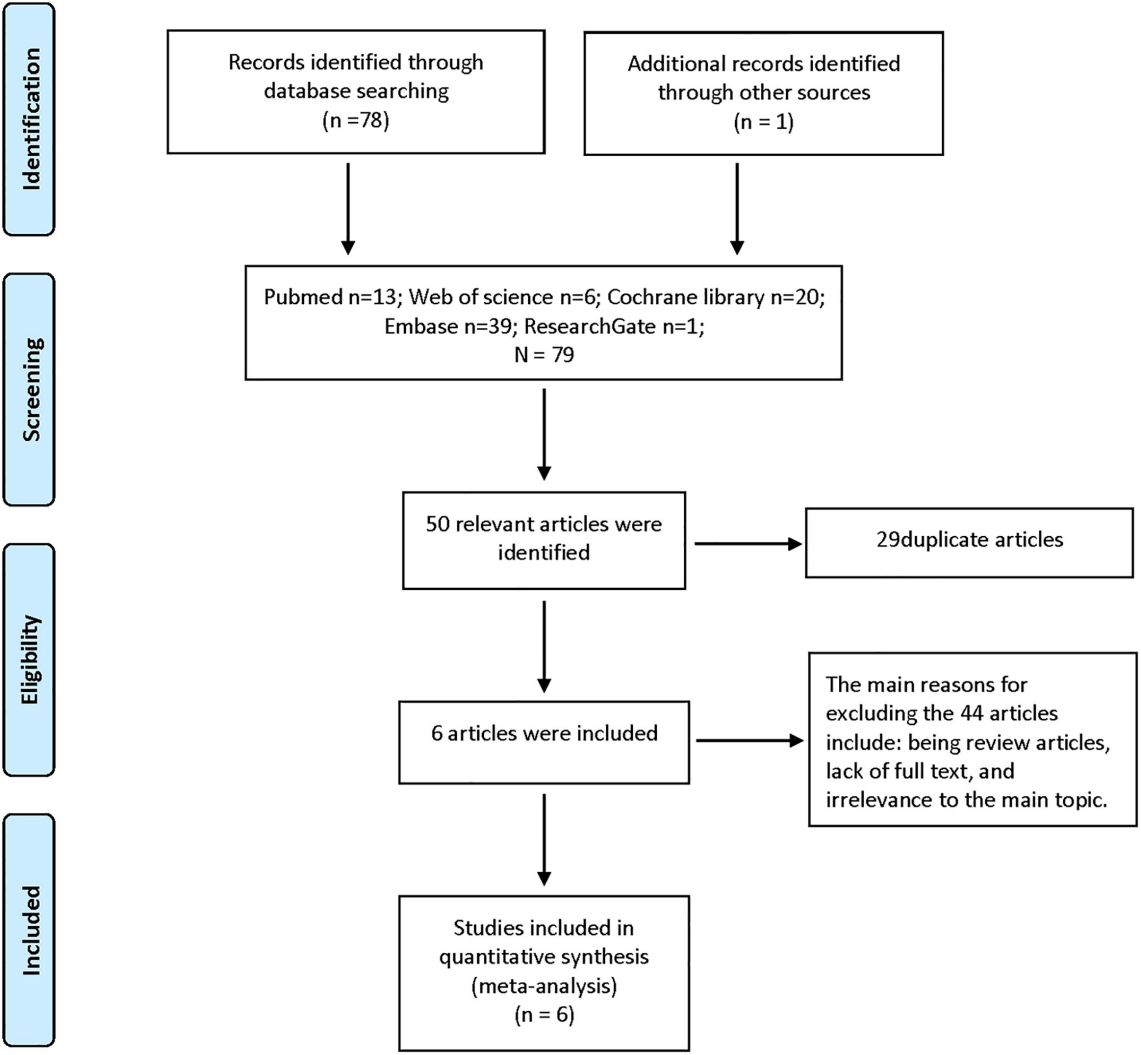
PRISMA flow diagram.

The authors each independently identified studies and trials that were potentially relevant. Following the completion of the study, we engaged in a collective discussion regarding the inclusion and exclusion criteria for each RCT. Excluded from the analysis were studies that did not meet the inclusion criteria or had unresolved discrepancies. Fig 1 illustrates the process of study selection.

### Quality assessment

The meta-analysis included all identified RCTs, irrespective of their quality scores. During the evaluation of RCT quality, criteria such as sequence generation, allocation concealment procedures, blinding, incomplete outcome data, selective outcome reporting, and other potential sources of bias were assessed. According to the Cochrane Handbook for Systematic Reviews of Interventions v.5.1.0 [20], the studies were qualitatively classified in adherence to the guidelines. Each study was assessed and categorized as having a low risk of bias (+), an unclear risk of bias (?), or a high risk of bias (-) based on the established quality assessment criteria. The authors reconciled their differences through discussion.

### Data extraction

In conducting the meta-analysis, we independently extracted the following data: (1) the name of the first author and the year of publication, (2) the study design, (3) the treatment therapy administered to patients, (4) the number of patients, (5) the male/female ratio, (6) follow-up period, and (7) eligibility criteria.We sought information about dropouts, withdrawals and other missing data and, if not reported, we contacted study authors for this information.

### Statistical analysis

The study outcomes were presented as odds ratios (ORs) with their corresponding 95% confidence intervals (CIs) for non-continuous variables. The mean difference (MD) and its 95% CI were employed for reporting continuous outcomes. The initial approach involved the use of the traditional meta-analysis method, which utilized the Cochrane Collaboration Review Manager software (RevMan v.5.1.0) to analyze the comparative effects. The I^2^ statistic was utilized to evaluate the amount of statistical heterogeneity. The presence of heterogeneity was considered when I^2^ was equal to or greater than 50%. A ’random effects’ model was utilized in the presence of heterogeneity, and a ’fixed-effects’ statistical model was employed when no obvious heterogeneity was observed.

## Results

### Characteristics of the individual studies

Six RCTs [21–26] involving 592 patients fulfilled the inclusion and exclusion criteria for the analysis. The literature selection process is illustrated in Fig 1 through a flow diagram. Table 1 provides a detailed list of the characteristics of the individual studies.

**Table 1:** Characteristics of RCTs included in the present meta-analysis.

Study	Design	Treatment		Sample size		Follow-up period	Eligibility criteria
		Alpha-blocker	Mirabegron	Alpha-blocker	Mirabegron		
Ahmed A 2023	RCT	Silodosin 8 mg	Mirabegron 50 mg	50	50	3weeks	Lower ureteric stones, 4–10 mm
Abdel MS 2023	RCT	Silodosin 8 mg	Mirabegron 50 mg	35	35	4weeks	Distal ureteral stones, less than 10 mm
Bayar G 2020	RCT	Silodosin 8 mg	Mirabegron 50 mg	35	29	4weeks	Proximal and distal ureteral stones, 4-10mm
Faridi MS-2024	RCT	Silodosin 8 mg	Mirabegron 50 mg	58	56	4weeks	Distal ureteric stone of size 5–10 mm
Morsy S 2022	RCT	Tamsulosin 0.4mg	Mirabegron 50 mg	60	68	30days	Distal ureteral stones, less than 10 mm
Samir M 2023	RCT	Silodosin 8 mg	Mirabegron 50 mg	59	57	4weeks	Distal ureteral stones,5-10mm

### Study quality

All five trials analyzed in the study were RCTs and were subsequently incorporated into the meta-analysis. As outlined in Table 2, the studies we identified exhibited a low risk of bias.

**Table 2:** ROB for included randomized controlled trials.

Study	Sequence generation	Allocation concealment	Blinding	Incomplete Outcome Data	Selective Outcome Reporting	Other Sources of Bias
Abdel MS 2023	**+**	+	+	+	+	+
Ahmed A 2023	+	+	+	+	+	+
Bayar G 2020	+	+	+	+	+	+
Faridi MS-2024	+	+	+	+	+	+
Morsy S 2022	+	+	+	-	+	+
Samir M 2023	+	+	+	+	+	+

ROB: risk of bias; +, indicates low risk of bias;?, unclear risk of bias; -, high risk of bias.

### Stone expulsion rate (SER)

**Fig 2:**
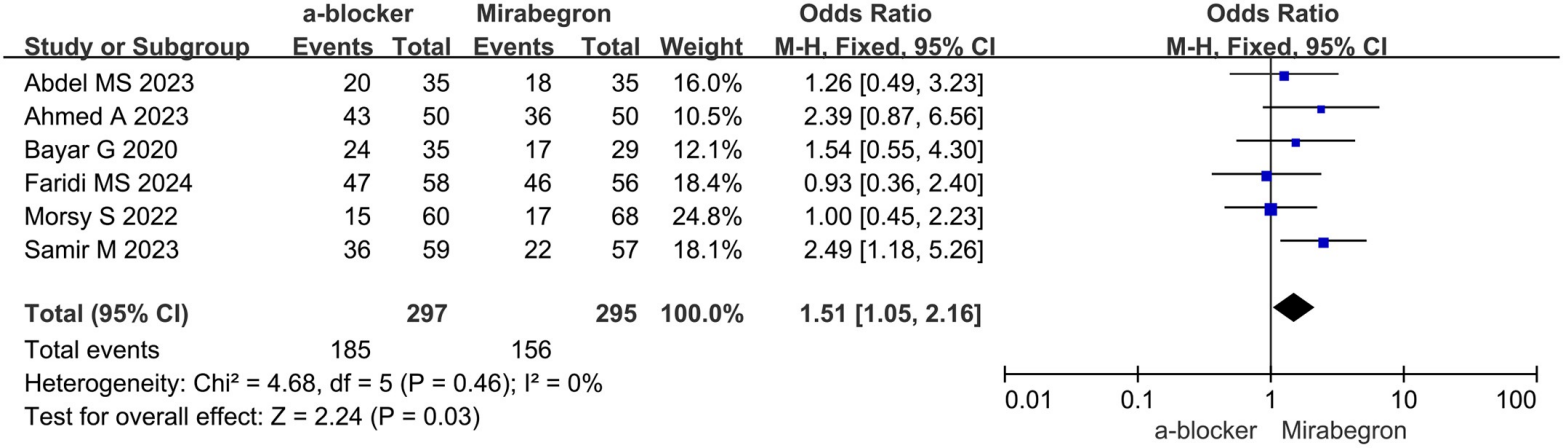
Stone expulsion rate in α-B group vs. MIR group.

The analysis of the SER was conducted using data from six studies, which included a total of 592 patients (297 in the α-B group and 295in the MIR group). Due to the lack of heterogeneity across the trials, we utilized a fixed effects model for the analysis. An odds ratio of 1.51 (95% CI: 1.05 to 2.16, p = 0.03) was reported in the forest plots. Among patients with UC, the α-B group exhibited a significantly higher SER than the MIR group, as revealed by the results. The analysis indicates that α-B therapy led to improved UC clearance (Fig 2).

### Stone expulsion time (SET)

**Fig 3:**
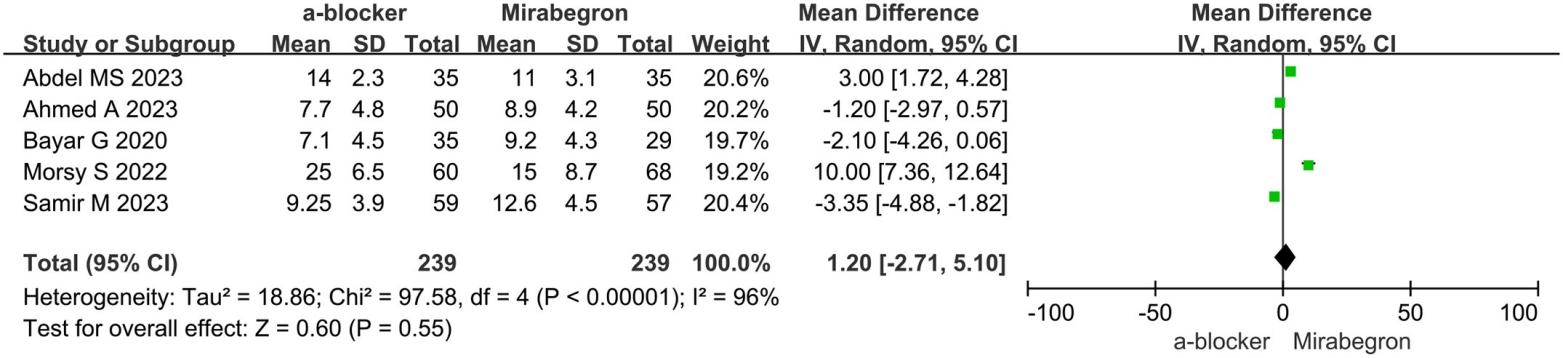
Time of expulsion in α-B group vs. MIR group.

The analysis of SET was based on data from five studies involving a total of 478 patients (239 in the α-B group and 239 in the MIR group). The trials exhibited heterogeneity (p<0.00001, I2 = 96%), requiring the application of a random effects model for the analysis. MD was 1.20 (95% CI, -2.71 to 5.10; p = 0.55), as shown in the forest plots. There was no notable disparity in SET between the α-B group and the MIR group for patients with UC, as revealed by the results (Fig 3).

### Pain episodes (PE)

**Fig 4:**
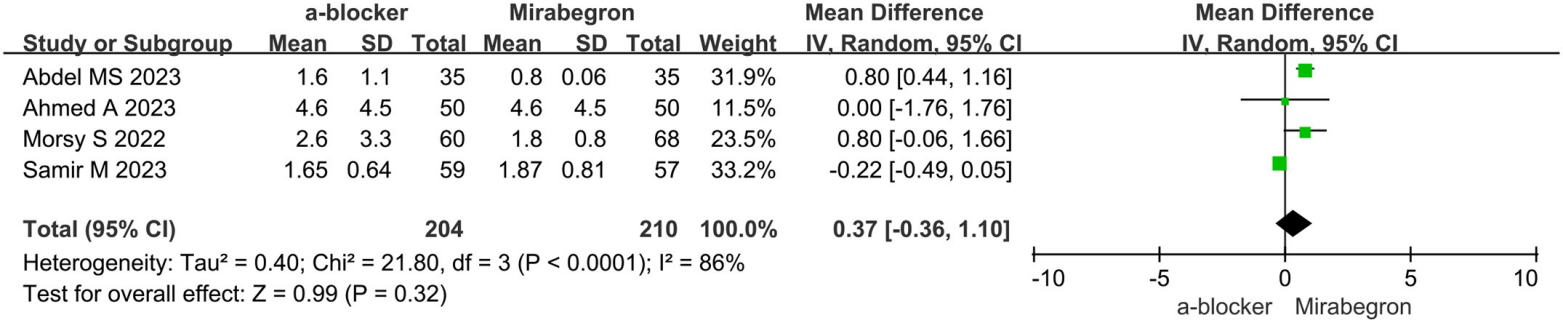
Pain eposides in α-B group vs. MIR group.

Data from five studies, encompassing 528 patients (262 in the α-B group and 266 in the MIR group), were employed in the analysis of PE. Given the heterogeneity observed among the trials (p<0.0001, I2 = 85%), the analysis employed a random effects model. After conducting our analysis, it became evident that the α-B had no effect on PE within the MIR group (Fig 4). This was evident in the forest plots, which depicted an MD of 0.36 (95% CI, -0.04 to 0.76; p = 0.07).

### Analgesic requirement

**Fig 5:**
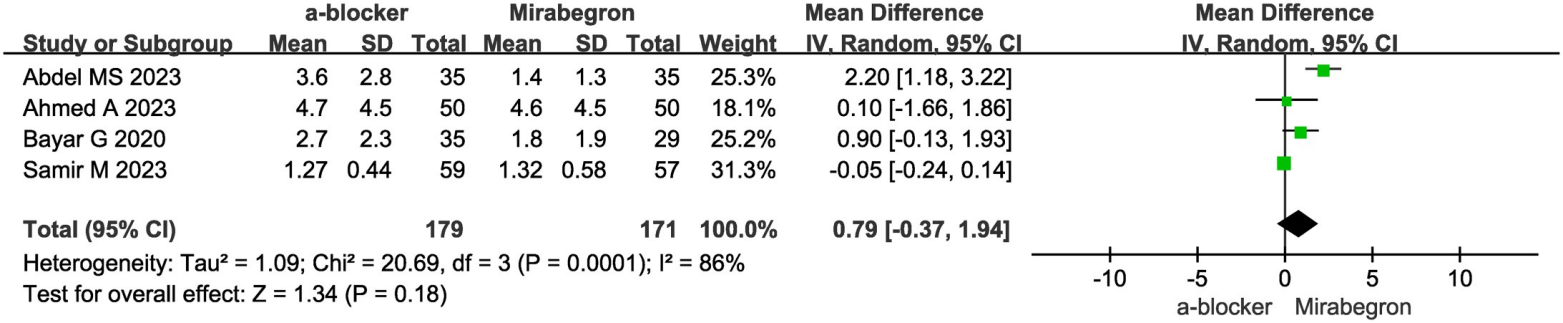
Analgesic requirement in α-B group vs. MIR group.

The analysis of analgesic requirements included data from four studies, encompassing 350 patients (179 in the α-B group and 171 in the MIR group). The presence of heterogeneity in the trials (p = 0.0001, I2 = 86%) required the utilization of a random effects model for the analysis. The forest plots indicated an MD of 0.79 (95% CI, -0.37 to 1.94; p = 0.18). Upon examination of the study, it becomes apparent that there was no notable discrepancy in analgesic needs between the α-B group and the MIR group, as depicted in Fig 5.

### Adverse events

No serious adverse effects were reported in any RCTs included in the meta-analysis. Headache, dizziness, orthostatic hypotension, retrograde ejaculation, and similar adverse events were commonly reported. Among all the included studies, two studies reported the incidence of stopped alpha blocker due to adverse effects. Bayar et al [23] reported two patients in silodosin group discontinued medication; Samir et al [25] reported one patient in silodosin group discontinued medication. Four studies with available data were included in our analysis. After analysis, it was found that α-B therapy was significantly associated with an increased incidence of adverse events, including orthostatic hypotension (OR 12.16, 95% CI 3.36 to 43.95, p = 0.0001), headache (OR 3.46, 95% CI 1.41 to 8.49, p = 0.007), and retrograde ejaculation (OR 16.30, 95% CI 5.87 to 45.31, p < 0.00001).While in the dizziness (OR 1.65, 95% CI 0.67 to 4.09, p = 0.28), it made no difference. (Table 3).

**Table 3:** The reported adverse events.

Outcome	Number of Studies	Number of Participants	Statistical method	Effect estimate	P value
Orthostatic hypotension	4	350	Odds Ratio (M-H, Fixed, 95% CI)	12.16 [3.36, 43.95]	P = 0.0001
Headache	3	286	Odds Ratio (M-H, Fixed, 95% CI)	3.46 [1.41, 8.49]	P = 0.007
Retrograde ejaculation	3	286	Odds Ratio (M-H, Fixed, 95% CI)	16.30 [5.87, 45.31]	P<0.00001
Dizziness	3	286	Odds Ratio (M-H, Fixed, 95% CI)	1.65 [0.67, 4.09]	P = 0.28

### Intervention of stent fixation and URS

The intervention treatments were initiated for patients who did not experience spontaneous passage of the stone or encountered worsening conditions and serious complications. Only two RCTs reported the intervention of stent fixation and URS. Ahmed et al [22] indicated that one patient in silodosin and two patients in mirabegron need stent fixation; six patients in silodosin and twelve patients in mirabegron need URS. Morsy et al [24] indicated that no patient in tamsulosin and four patients in mirabegron need stent fixation; four patients in tamsulosin and three patients in mirabegron need URS. There is nonsignificant difference between the α-B group and MIR group.

## Discussion

UC comprises 22% of nephrolithiasis cases, with distal UC accounting for 68% of these cases [12, 27]. The incidence of ureteral calculi has steadily increased in recent decades, resulting in significant social and economic burdens [28]. Recurrence is common, with as many as 50% of patients experiencing recurrence within five years [29]. In emergency departments, MET has been extensively utilized for patients with ureteric colic, particularly for small UCs located in the distal region [30]. The clinical use of both selective α-B and MIR is aimed at improving the passage of UC in patients [5].

The activation of α-ARs is implicated in causing contraction in the mammalian ureter, while the activation of β-ARs is associated with relaxation, thus emphasizing the involvement of the sympathetic nervous system in ureteral physiology [31, 32]. There are three different subtypes of α-AR: α1A, α1B, and α1D [33]. The distribution of alpha1-AR in the human ureter reveals a greater prevalence of the alpha-1A and alpha-1D subtypes in the distal ureter and ureterovesical junction, as opposed to the proximal and middle ureters [34]. The enhancement of ureteral contraction and increase in ureteral peristalsis is a result of the stimulation of alpha1-AR [35]. First-dose hypotension, syncope, dizziness, headache, and retrograde ejaculation may occur due to the vasodilation and relaxation of vascular smooth muscle caused by selective alpha-1 blockers [36]. The β-ARs are categorized into β1-, β2-, and β3-AR subtypes. All β-AR subtypes found in the ureter are expressed in both smooth muscles and the urothelium [15]. MIR, the first β3-adrenoceptor agonist, induces relaxation by modulating the function of the urinary tract epithelium. Consequently, it indirectly impacts muscle tone, inhibits ureteral smooth muscle contraction, and dilates the ureter [37]. Research has indicated that MIR primarily leads to adverse effects such as hypertension, nasopharyngitis, urinary tract infections, tachycardia, headache, back pain, dizziness, and joint pain. Except for nasopharyngitis, the incidence of other adverse effects did not differ from that of the placebo [38, 39].

A meta-analysis by Amer on MET revealed that α-B increased the SER of distal stones larger than 5 mm yet did not exhibit the same effect for smaller and more proximally located stones. Furthermore, α-B was connected to reduced expulsion durations and elevated side effect occurrences [40]. Another meta-analysis performed by Sridharan revealed that tamsulosin and silodosin notably improved the SER of UC≥5 mm, reduced the time for stone expulsion, and lessened the frequency of pain attacks. Nevertheless, they were also related to a heightened risk of adverse events compared to the placebo [41]. The latest meta-analysis published by Cai indicated that, compared to a placebo. However, MIR cannot reduce SET, but it can markedly enhance the rate of stone expulsion, diminish PE, and exhibit a similar incidence of adverse events [19].

Our meta-analysis aimed to assess the effectiveness and safety of α-B in comparison to MIR as a MET for UC. In the analysis, a statistically significant difference in SER was observed between the α-B and MIR groups, with the former showing a higher rate (p = 0.03). The α-B group and the MIR group did not show significant differences in terms of SET (p = 0.55), PE (p = 0.07), or analgesic requirements (p = 0.18). In terms of adverse events, the pooled analysis showed that the group receiving α-B had a significantly greater incidence of orthostatic hypotension (p = 0.0001), headache (p = 0.007), and retrograde ejaculation (p < 0.00001). No severe adverse effects were reported in any of the RCTs included in the meta-analysis.

The RCTs included in our meta-analysis were articles indexed in the Science Citation Index and showed a low risk of bias. However, despite the high quality of the included studies, our research has several limitations. Firstly, our meta-analysis was restricted to five RCTs due to the scarcity of pertinent original studies. Additionally, the heterogeneity in certain outcomes may be attributed to differences in the location, size, and composition of stones, as well as variations in the types of α-B and follow-up durations among the included studies. Additionally, efficacy outcomes and safety data were reported with incomplete or insufficient detail in several studies. This meta-analysis is pivotal in assessing the effectiveness of α-B in comparison to MIR for treating UC, even when taking into account the heterogeneity among individual studies. Therefore, there is an urgent need for additional high-quality RCTs with longer follow-up periods and larger sample sizes to conclusively establish the safety and effectiveness of α-B and MIR in managing UC.

## Conclusions

In conclusion, our meta-analysis indicates that α-B is more effective to MIR in treating UC, particularly in significantly enhancing the SER. Regarding safety, the occurrence of adverse events was higher for α-B than for MIR.

## Supporting information

S1 Checklist Prisma checklist for reporting systematic reviews. (DOCX)

S1 File Supporting information including the data extraction word file, the quality assessment figure, evaluation article. (ZIP)

## Abbreviations

AR: adrenergic receptors
α-B: alpha-blockers
MET: medical expulsive therapy
MIR: mirabegron
PE: pain episodes
PNL: percutaneous nephrolithotomy
PDE: phosphodiesterase
RCTs: randomized controlled trials
SWL: shockwave lithotripsy
SER: stone expulsion rate
SET: stone expulsion time
UC: ureteral calculi
