# Supplementary material for: Evaluating the safety and effectiveness of α-blockers versus mirabegron for medical expulsive therapy in ureteral calculi: A Systematic review and meta-analysis
Source: PLoS One. 2024 Dec 27;19(12):e0315328. doi: 10.1371/journal.pone.0315328 (PMC11676830; doi:10.1371/journal.pone.0315328)
Supplement: S1 File — (ZIP) [file pone.0315328.s002.zip › Supporting information including the data extraction word file, the quality assessment figure, evaluation article/Characteristics of included studies [ordered by study ID].docx]

1.Faridi MS-2024

| Study characteristics | | |
| --- | --- | --- |
| Methods | **Study design:** a prospective, cohort, double blind, randomised study  Duration of study: 4weeks | |
| Partiipants | **Setting:** hospital  **Country**:India  **Number of participants:** 58 silodosin ; 56 mirabegron  **Age, mean (SD) years**: silodosin 32.5 (9.67 )years, mirabegron 33.12 (7.84) years  **Sex:** silodosin 39M/19 F; mirabegron 36 M/20 F  **Inclusion criteria:** patients aged ≥18 years,with distal ureteric stone of size 5–10 mm in maximum diameter, diagnosed on ultrasonography(USG), of the kidney, ureter, or bladder (KUB),or non-contrast computed tomography KUB.  **Exclusion criteria:**active urinary tract infection, severe hydronephrosis, bilateral or multiple unilateral ureteric stones, solitary kidney, renal insuffciency, uncontrolled hypertension (systolic blood pressure ≥180 mmHg, diastolic blood pressure ≥110 mmHg), previous ureter and urinary bladder surgery, patient on alpha-blocker or anticholinergic to lower urinary tract symptoms, hepatic dysfunction, ureteric strictures, pregnancy, and those who opted out of the study | |
| Interventions | **Intervention 1:**silodosin 8 mg once daily  I**ntervention 2:**mirabegron 50 mg once daily  All the patients were advised to take 2.5 to 3 litres of water daily and a diclofenac 50 mg tablet orally for pain episodes.  **Follow-up:** The drugs were continued until stone expulsion or 4 weeks. | |
| Outcomes | **Primary:** the stone expulsion rate  **Secondary:** stone expulsion interval, rates of interventions, pain episodes and adverse events | |
| Funding | Not reported | |
| Declarations of interest | Quote: "The authors declare no conﬂicts of interest.” | |
| Notes | None | |
| **Risk of bias** | | |
| **Bias** | **Authors'judgement** | **Support for judgement** |
| Random sequence genera-  tion (selection bias) | Low risk | Quote: "Patients were randomised into 2 groups based on sequentially numbered, opaque, sealed envelope (SNOSE) technique."  Comment: study judged at low risk of bias. |
| Allocation concealment  (selection bias) | Low risk | Quote: "Patients were randomised into 2 groups based on sequentially numbered, opaque, sealed envelope (SNOSE) technique".  Comment: study judged at low risk of bias. |
| Blinding of participants  and personnel (perfor-  mance bias)  All outcomes | Low risk | Quote: "This was the prospective, cohort, double blind, randomised study "  Comment: Though the study did not specify the method of blinding. Blinding of participants and personnel was conducted.Review authors judged at low risk of bias. |
| Blinding of outcome as-  sessment (detection bias) All outcomes | Low risk | Comment: Whether the application of blinding method will not affect the outcome or outcome measurment,study judged at low risk of bias. |
| Incomplete outcome data  (attrition bias)  All outcomes | Low risk | 3patients in both group lost to follow-up. Discontinuation of the medication occurred in 6 patients: 4 in the silodosin group (retrograde ejaculation, hypotension) and 2 in the mirabegron group (hypertension).  Comment: fewer than 20% of participants dropped out or withdrew, and the study author performed ITT analysis; study judged at low risk of attrition bias. |
| Selective reporting (re-  porting bias) | Low risk | Comment: all of the study's pre-specified outcomes were reported in the result; study judged at low risk of attrition bias. |
| Other bias | Low risk | Comment: we did not find any methodological issues that might directly lead to a risk of bias. |

2.Ahmed A 2023

| Study characteristics | | |
| --- | --- | --- |
| Methods | **Study design:** A Prospective, Randomize, Randomized, Comparative Study  **Duration of study:** 3weeks | |
| Participants | **Setting:** outpatient clinic  **Country**: Egypt  **Number of participants:** 50 silodosin ; 50 mirabegron  **Age, mean (SD) years**: silodosin 38.5(13.3)years, mirabegron39.3(14.4) years  **Sex:** silodosin 34M/16F; mirabegron 37 M/13F  **Inclusion criteria:** .patients of both Sexs who had a single, unilateral lower ureteric stone(beneath the sacroiliac joint) between 4 and 10 mm in size.  **Exclusion criteria:** Solitary kidney, bilateral ureteric stones, active urinary tract infection, renal insufficiency, moderate or severe hydronephrosis, ureteral obstruction distal to the stone, prior history of ipsilateral ureter surgery, pregnant or lactating women, and comorbidities precluding the use of study medications. | |
| Interventions | **Intervention 1:** a daily dose of 8 mg of silodosin  I**ntervention 2:** a daily dose of 50 mg of mirabegron  Patients would receive diclofenac potassium as an analgesic for the control of ureteral colic pain.  **Follow-up:** The medications would be continued until expulsion of the stone, up to a maximum of 3 weeks. | |
| Outcomes | **Primary:** time to stone expulsion  **Secondary:** rate of stone expulsion,frequency and severity of loin pain, frequency and cumulative dose of analgesics, unplanned hospital admission due to infected hydronephrosis, side effects of the study medications, and the need and type of intervention. | |
| Funding | Quote: "This research did not receive any specific grant from funding agencies in the public, commercial, or not-for-profit sectors.”  Comment：no sponsors have influenced the report of outcomes. | |
| Declarations of interest | Quote: "The authors have no financial interest to declare regarding the content of this article.” | |
| Notes | None | |
| **Risk of bias** | | |
| **Bias** | **Authors'judgement** | **Support for judgement** |
| Random sequence genera-  tion (selection bias) | Low risk | Quote: "The patients were randomly divided into three equal groups using a computer-generated random number table prepared by a statistician who was not part of the research team."  Comment: study judged at low risk of bias. |
| Allocation concealment  (selection bias) | Low risk | Quote: "The patients were randomly divided into three equal groups using a computer-generated random number table prepared by a statistician who was not part of the research team."  Comment: study judged at low risk of bias. |
| Blinding of participants  and personnel (perfor-  mance bias)  All outcomes | Low risk | Comment: Though insufficient information reported blinding, the outcome or the outcome measurement was unlikely to be influenced by the lack of blinding, study judged at low risk of bias. |
| Blinding of outcome as-  sessment (detection bias) All outcomes | Low risk | Comment: Though insufficient information reported blinding, the outcome or the outcome measurement was unlikely to be influenced by the lack of blinding, study judged at low risk of bias. |
| Incomplete outcome data  (attrition bias)  All outcomes | Low risk | 2 patients were lost on follow up in silodosin group; 1 patient were lost on follow up in mirabegron group  Comment: fewer than 20% of participants dropped out or withdrew, study judged at low risk of attrition bias. |
| Selective reporting (re-  porting bias) | Low risk | Comment: all of the study's pre-specified outcomes were reported in the result; study judged at low risk of attrition bias. |
| Other bias | Low risk | Comment: we did not find any methodological issues that might directly lead to a risk of bias. |

3**.Abdel MS 2023**

| Study characteristics | | |
| --- | --- | --- |
| Methods | **Study design:** a prospective, randomized study  **Duration of study:** 4weeks | |
| Participants | **Setting:** outpatient clinic  **Country**: Egypt  **Number of participants:** 35 silodosin ; 35 mirabegron  **Age, mean (SD) years**: silodosin 36.65(6.81)years, mirabegron38.62 (7.88) years  **Sex:** silodosin 23M/12F; mirabegron 22 M/13F  **Inclusion criteria:** .patients over 18 years old with a single radioopaque stone≤10 mm located in the distal ureter.  **Exclusion criteria:** single functioning kidney, impaired renal function, multiple or bilateral and radiolucent ureteric stones, severe persistent pain, urinary tract infection, severe hydronephrosis (grade IV according to SFU grading system), uncontrolled hypertension, pregnancy, anatomical abnormalities, current use of alpha-blockers, and previous ureteral surgery. | |
| Interventions | **Intervention 1:** a daily dose of 8 mg of silodosin  I**ntervention 2:** a daily dose of 50 mg of mirabegron  All the patients were advised to take 2.5 to 3 litres of water daily and a diclofenac 50 mg tablet orally for pain episodes. All patients were instructed to consume a minimum of 2500–3000 mL of water daily and strain their urine to detect any potential stones.  **Follow-up:** The follow-up continued until the stone passed spontaneously or until the treatment was discontinued after 4 weeks. | |
| Outcomes | **Primary:** the rate of stone expulsion  **Secondary:** the time it took for the stones to be expelled, the number of pain attacks, the amount of additional analgesia required, and the adverse effects associated with the medication | |
| Funding | Quote: "Open access funding provided by The Science, Technology & Innovation Funding Authority (STDF) in cooperation with The Egyptian Knowledge Bank (EKB).”  Comment：the sponsor of the study had no role in the conduct of the analysis or drafting of the report. | |
| Declarations of interest | Quote: "There is no conflict of interest” | |
| Notes | None | |
| **Risk of bias** | | |
| **Bias** | **Authors'judgement** | **Support for judgement** |
| Random sequence generation (selection bias) | Low risk | Quote: "The patients were randomly divided into three equal groups using a computer-generated random number table prepared by a statistician who was not part of the research team."  Comment: study judged at low risk of bias. |
| Allocation concealment  (selection bias) | Low risk | Quote: "The patients were randomly divided into three equal groups using a computer-generated random number table prepared by a statistician who was not part of the research team.".  Comment: study judged at low risk of bias. |
| Blinding of participants  and personnel (performance bias)  All outcomes | Low risk | Comment: It is oral treatments,whether the application of blinding method will not affect the outcome or outcome measurement,study judged at low risk of bias. |
| Blinding of outcome assessment (detection bias) All outcomes | Low risk | Comment: It is oral treatments,whether the application of blinding method will not affect the outcome or outcome measurement,study judged at low risk of bias. |
| Incomplete outcome data  (attrition bias)  All outcomes | Low risk | 2 patients were lost on follow up in silodosin group; 1 patient were lost on follow up in mirabegron group  Comment: fewer than 20% of participants dropped out or withdrew, study judged at low risk of attrition bias. |
| Selective reporting (re-  porting bias) | Low risk | Comment: all of the study's pre-specified outcomes were reported in the result; study judged at low risk of attrition bias. |
| Other bias | Low risk | Comment: we did not find any methodological issues that might directly lead to a risk of bias. |

4.Bayar G 2020

| Study characteristics | | |
| --- | --- | --- |
| Methods | **Study design:** a prospective, randomized multicentric study  **Duration of study:** 4weeks | |
| Participants | **Setting:** Multi-center  **Country**: Turkey  **Number of participants:** 54 silodosin ; 56 mirabegron  **Age, mean (SD) years**: silodosin 40(15)years, mirabegron43(13.3) years  **Sex:** silodosin 40M/14F; mirabegron 48 M/8F  **Inclusion criteria:** Patients who had ureter stones in size between 4 and 10 mm.  **Exclusion criteria:** solitary kidney, bilateral ureter stone, stones bigger than 10 mm, urinary tract infection, acute azotemia, use of another alpha-blocker or anticholinergic for lower urinary tract symptoms, residual stone after any procedure (shock wave lithotripsy, ureterolithotripsy, ureterolithotomy, etc.), patients with uncontrolled hypertension or hepatic dysfunction, pregnancy, and childhood. | |
| Interventions | **Intervention 1:** a daily dose of 8 mg of silodosin  I**ntervention 2:** a daily dose of 50 mg of mirabegron  **Follow-up:** Patients were followed weekly for 4 weeks by ultrasonography and/or KUB, if patients could not see stones in their urine. | |
| Outcomes | **Primary:** the rate of stone expulsion  **Secondary:**stone expulsion interval, total required analgesic dosage of the patients and adverse events | |
| Funding | none | |
| Declarations of interest | Quote: "Authors declare no confict of interest.” | |
| Notes | None | |
| **Risk of bias** | | |
| **Bias** | **Authors'judgement** | **Support for judgement** |
| Random sequence generation (selection bias) | Low risk | Quote: "For randomization, we used a block-randomized scheme designed by an experienced statistician."  Comment: study judged at low risk of bias. |
| Allocation concealment  (selection bias) | Low risk | Quote: "For randomization, we used a block-randomized scheme designed by an experienced statistician."  Comment: study judged at low risk of bias. |
| Blinding of participants  and personnel (performance bias)  All outcomes | Low risk | Comment: It is oral treatments,whether the application of blinding method will not affect the outcome or outcome measurement,study judged at low risk of bias. |
| Blinding of outcome assessment (detection bias) All outcomes | Low risk | Comment: It is oral treatments,whether the application of blinding method will not affect the outcome or outcome measurement,study judged at low risk of bias. |
| Incomplete outcome data  (attrition bias)  All outcomes | Low risk | 4 patients were lost on follow up in silodosin group; 2patient were lost on follow up in mirabegron group.Discontinuation of the medication occurred in 3 patients: 2 in the silodosin group (hypotension) and 1 in the mirabegron group (hypertension).  Comment: fewer than 20% of participants dropped out or withdrew, study judged at low risk of attrition bias. |
| Selective reporting (reporting bias) | Low risk | Comment: all of the study's pre-specified outcomes were reported in the result; study judged at low risk of attrition bias. |
| Other bias | Low risk | Comment: we did not find any methodological issues that might directly lead to a risk of bias. |

5.Morsy S 2022

| Study characteristics | | |
| --- | --- | --- |
| Methods | **Study design:** a prospective randomized controlled study  **Duration of study:** 30 days | |
| Participants | **Setting:** outpatient clinics  **Country**: Egypt  **Number of participants:** 25tamsulosin ; 25mirabegron  **Age, mean (SD) years**: tamsulosin 37.48(4.56)years, mirabegron47.28(4.05) years  **Sex:** tamsulosin 22M/3F; mirabegron 16 M/9F  **Inclusion criteria:** patients with a unilateral distal ureteral stone <10 mm in maximum length in computed tomography [CT] scan)  **Exclusion criteria:** the grade 3 or 4 ureterohydronephrosis, renal impairment, solitary kidney, intractable renal colic (not relieved by commonly used analgesia), multiple ureteral stones, pregnancy, prior ureter and bladder operations, uncontrolled systemic hypertension, existing use of Ca-channel or α-blockers, and pediatric age-group. | |
| Interventions | **Intervention 1:**tamsulosin HCL 0.4 mg cap once-daily+ diclofenac Na 100 mg tab (to be gained only during colic episodes)  I**ntervention 2:** mirabegron 50 mg once-daily + diclofenac Na 100 mg tab (to be gained only during colic episodes)  **Follow-up:** patients were observed for 30 days | |
| Outcomes | **Primary:** the rate of stone expulsion and stone expulsion time  **Secondary:**colic episodes and drug adverse effect | |
| Funding | Quote: " This manuscript did not receive any funding.” | |
| Declarations of interest | Quote: "The authors have no conflicts of interest to declare.” | |
| Notes | None | |
| **Risk of bias** | | |
| **Bias** | **Authors'judgement** | **Support for judgement** |
| Random sequence generation (selection bias) | Low risk | Quote: "patients were randomly assigned to mirabegron, tamsulosin HCL, and/or diclofenac Na groups by applying the volatile numbers created via RAND-function in Excel."  Comment: study judged at low risk of bias. |
| Allocation concealment  (selection bias) | Low risk | Quote: "Each patient received a sealed envelope for his modality of treatment."  Comment: study judged at low risk of bias. |
| Blinding of participants  and personnel (performance bias)  All outcomes | Low risk | Quote “Throughout the study, the investigators and patients were masked about the treatment type. ”  Comment: study judged at low risk of bias. |
| Blinding of outcome assessment (detection bias) All outcomes | Low risk | Quote “Throughout the study, the investigators and patients were masked about the treatment type. ”  Comment: study judged at low risk of bias. |
| Incomplete outcome data  (attrition bias)  All outcomes | High risk | 1patients were lost on follow up in tamsulosin group; 2patient were lost on follow up in mirabegron group.Discontinuation of the medication occurred in 7 patients: 4 in the tamsulosin group (non compliance) and 3 in the mirabegron group (nasal congestion ,hypertension).  Comment: more than 20% of participants dropped out or withdrew, study judged at high risk of attrition bias. |
| Selective reporting (reporting bias) | Low risk | Comment: all of the study's pre-specified outcomes were reported in the result; study judged at low risk of attrition bias. |
| Other bias | Low risk | Comment: we did not find any methodological issues that might directly lead to a risk of bias. |

1. Samir M 2023

| Study characteristics | | |
| --- | --- | --- |
| Methods | **Study design:** a prospective randomized controlled study  **Duration of study:** 4weeks | |
| Participants | **Setting:** hospital  **Country**: Egypt  **Number of participants:** 59 silodosin ; 57 mirabegron  **Age, mean (SD) years**: silodosin 38.6(11.1)years, mirabegron 39.5(10.5) years  **Sex:** silodosin 30M/29F; mirabegron 27M/30F  **Inclusion criteria:** Patients aged 18–60years old complaining of a single radiopaque ureteral stone in the distal part (below the sacroiliac joint) and of 5–10mm size.  **Exclusion criteria:** Patients with solitary kidney, bilateral ureteric stones, renal impairment, urinary tract infection (UTI), severe renal colic, pregnancy, severe hydronephrosis, uncontrolled hypertension, any urologic anomalies or previous history of ureteral surgery. | |
| Interventions | **Intervention 1:** a daily dose of 8 mg of silodosin  I**ntervention 2:** a daily dose of 50 mg of mirabegron  **Follow-up:** The treatment was continued until stone passage or maximally for 4weeks. | |
| Outcomes | **Primary:** the stone expulsion rate (SER)  **Secondary:** stone expulsion time, side effects due to the used drugs, hospital visits number for pain, and amount of analgesic taken. | |
| Funding | Quote: "The author(s) received no financial support for the research, authorship, and/or publication of this article.” | |
| Declarations of interest | Quote: "The author(s) declared no potential conflicts of interest with respect to the research, authorship, and/or publication of this article.” | |
| Notes | None | |
| **Risk of bias** | | |
| **Bias** | **Authors'judgement** | **Support for judgement** |
| Random sequence generation (selection bias) | Low risk | Quote: "One hundred eighty patients who fulfilled the inclusion criteria were randomly divided using computer generated method into three equal groups of 60 patients."  Comment: study judged at low risk of bias. |
| Allocation concealment  (selection bias) | Low risk | Quote: "One hundred eighty patients who fulfilled the inclusion criteria were randomly divided using computer generated method into three equal groups of 60 patients."  Comment: study judged at low risk of bias. |
| Blinding of participants  and personnel (performance bias)  All outcomes | Low risk | Quote: "The study was designed as a double-blind one."  Comment: It is oral treatments,whether the application of blinding method will not affect the outcome or outcome measurement,study judged at low risk of bias. |
| Blinding of outcome assessment (detection bias) All outcomes | Low risk | Quote: "The study was designed as a double-blind one."  Comment: It is oral treatments,whether the application of blinding method will not affect the outcome or outcome measurement,study judged at low risk of bias. |
| Incomplete outcome data  (attrition bias)  All outcomes | Low risk | 3patient were lost on follow up in mirabegron group;1 patient stopped treatment due to side effects in the silodosin group.  Comment: fewer than 20% of participants dropped out or withdrew, study judged at low risk of attrition bias. |
| Selective reporting (reporting bias) | Low risk | Comment: all of the study's pre-specified outcomes were reported in the result; study judged at low risk of attrition bias. |
| Other bias | Low risk | Comment: we did not find any methodological issues that might directly lead to a risk of bias. |
