# Supplementary material for: Evaluating the safety and effectiveness of α-blockers versus mirabegron for medical expulsive therapy in ureteral calculi: A Systematic review and meta-analysis
Source: PLoS One. 2024 Dec 27;19(12):e0315328. doi: 10.1371/journal.pone.0315328 (PMC11676830; doi:10.1371/journal.pone.0315328)
Supplement: S1 File — (ZIP) [file pone.0315328.s002.zip › Supporting information including the data extraction word file, the quality assessment figure, evaluation article/Data extraction .docx]

**Supplementary materials and data**

Alpha-blocker versus Mirabegron

| Stone expulsion rate | a-blocker | | Mirabegron | |
| --- | --- | --- | --- | --- |
| Abdel MS 2023 | 20 | 35 | 18 | 35 |
| Ahmed A 2023 | 43 | 50 | 36 | 50 |
| Bayar G 2020 | 24 | 35 | 17 | 29 |
| Faridi MS 2024 | 47 | 58 | 46 | 56 |
| Morsy S 2022 | 15 | 60 | 17 | 68 |
| Samir M 2023 | 36 | 59 | 22 | 57 |

| Time of expulsion | a-blocker | | | Mirabegron | | |
| --- | --- | --- | --- | --- | --- | --- |
| Abdel MS 2023 | 14 | 2.3 | 35 | 11 | 3.1 | 35 |
| Ahmed A 2023 | 7.7 | 4.8 | 50 | 8.9 | 4.2 | 50 |
| Bayar G 2020 | 7.1 | 4.5 | 35 | 9.2 | 4.3 | 29 |
| Morsy S 2022 | 25 | 6.5 | 60 | 15 | 8.7 | 68 |
| Samir M 2023 | 9.25 | 3.9 | 59 | 12.6 | 4.5 | 57 |

| Pain episodes | a-blocker | | | Mirabegron | | |
| --- | --- | --- | --- | --- | --- | --- |
| Abdel MS 2023 | 1.6 | 1.1 | 35 | 0.8 | 0.06 | 35 |
| Ahmed A 2023 | 4.6 | 4.5 | 50 | 4.6 | 4.5 | 50 |
| Faridi MS 2024 | 2.34 | 0.2 | 58 | 1.94 | 0.18 | 56 |
| Morsy S 2022 | 2.6 | 3.3 | 60 | 1.8 | 0.8 | 68 |
| Samir M 2023 | 1.65 | 0.64 | 59 | 1.87 | 0.81 | 57 |

| Analgesic requirement | a-blocker | | | Mirabegron | | |
| --- | --- | --- | --- | --- | --- | --- |
| Abdel MS 2023 | 3.6 | 2.8 | 35 | 1.4 | 1.3 | 35 |
| Ahmed A 2023 | 4.7 | 4.5 | 50 | 4.6 | 4.5 | 50 |
| Bayar G 2020 | 2.7 | 2.3 | 35 | 1.8 | 1.9 | 29 |
| Samir M 2023 | 1.27 | 0.44 | 59 | 1.32 | 0.58 | 57 |

| hypotension | a-blocker | | Mirabegron | |
| --- | --- | --- | --- | --- |
| Abdel MS 2023 | 11 | 35 | 0 | 35 |
| Ahmed A 2023 | 10 | 50 | 0 | 50 |
| Bayar G 2020 | 2 | 35 | 0 | 29 |
| Samir M 2023 | 3 | 59 | 1 | 57 |

| Headache | a-blocker | | Mirabegron | |
| --- | --- | --- | --- | --- |
| Abdel MS 2023 | 12 | 35 | 3 | 35 |
| Ahmed A 2023 | 6 | 50 | 2 | 50 |
| Samir M 2023 | 3 | 59 | 2 | 57 |

| Dizziness | a-blocker | | Mirabegron | |
| --- | --- | --- | --- | --- |
| Abdel MS 2023 | 5 | 35 | 1 | 35 |
| Ahmed A 2023 | 4 | 50 | 2 | 50 |
| Samir M 2023 | 4 | 59 | 5 | 57 |

| Retrograde ejaculation | a-blocker | | Mirabegron | |
| --- | --- | --- | --- | --- |
| Abdel MS 2023 | 17 | 35 | 4 | 35 |
| Ahmed A 2023 | 11 | 50 | 0 | 50 |
| Samir M 2023 | 19 | 59 | 0 | 57 |

| Stent fixation | a-blocker | | Mirabegron | |
| --- | --- | --- | --- | --- |
| Ahmed A 2023 | 1 | 50 | 2 | 50 |
| Morsy S 2022 | 0 | 60 | 4 | 68 |
|  |  |  |  |  |
|  |  |  |  |  |

**Table 1. Characteristics of RCTs included in the present meta-analysis**

| Study | Design | Treatment | | Sample size | | Follow-up period | Eligibility criteria |
| --- | --- | --- | --- | --- | --- | --- | --- |
|  |  | Experimental | Control | Experimental | Control |  |  |
| Bayar G 2020 | RCT | Mirabegron 50 mg | Silodosin 8 mg | 29 | 35 | 4weeks | Proximal and distal ureteral stones, 4-10mm |
| Morsy S 2022 | RCT | Mirabegron 50 mg | Tamsulosin 0.4mg | 68 | 60 | 4weeks | Distal ureteral stones, less than 10 mm |
| Samir M 2023 | RCT | Mirabegron 50 mg | Silodosin 8 mg | 57 | 59 | 4weeks | Distal ureteral stones,5-10mm |
| Ahmed A 2023 | RCT | Mirabegron 50 mg | Silodosin 8 mg | 50 | 50 | 3weeks | Lower ureteric stones, 4-10 mm |
| Abdel MS 2023 | RCT | Mirabegron 50 mg | Silodosin 8 mg | 35 | 35 | 4weeks | Distal ureteral stones, less than 10 mm |

**Table 2. ROB for included randomized controlled trials**

| Study | Sequence generation | Allocation concealment | Blinding | Incomplete Outcome Data | Selective Outcome Reporting | Other Sources of Bias |
| --- | --- | --- | --- | --- | --- | --- |
| Abdel MS 2023 | **+** | + | + | + | ? | + |
| Ahmed A 2023 | + | + | + | + | + | ? |
| Bayar G 2020 | + | + | + | + | ? | ? |
| Morsy S 2022 | + | + | + | ? | + | + |
| Samir M 2023 | + | + | + | + | + | ? |

ROB: risk of bias; +, indicates low risk of bias; ?, unclear risk of bias; -, high risk of bias.
