# Supplementary material for: Evaluating the safety and effectiveness of α-blockers versus mirabegron for medical expulsive therapy in ureteral calculi: A Systematic review and meta-analysis
Source: PLoS One. 2024 Dec 27;19(12):e0315328. doi: 10.1371/journal.pone.0315328 (PMC11676830; doi:10.1371/journal.pone.0315328)
Supplement: S1 File — (ZIP) [file pone.0315328.s002.zip › Supporting information including the data extraction word file, the quality assessment figure, evaluation article/Risk of bias summary.pdf]

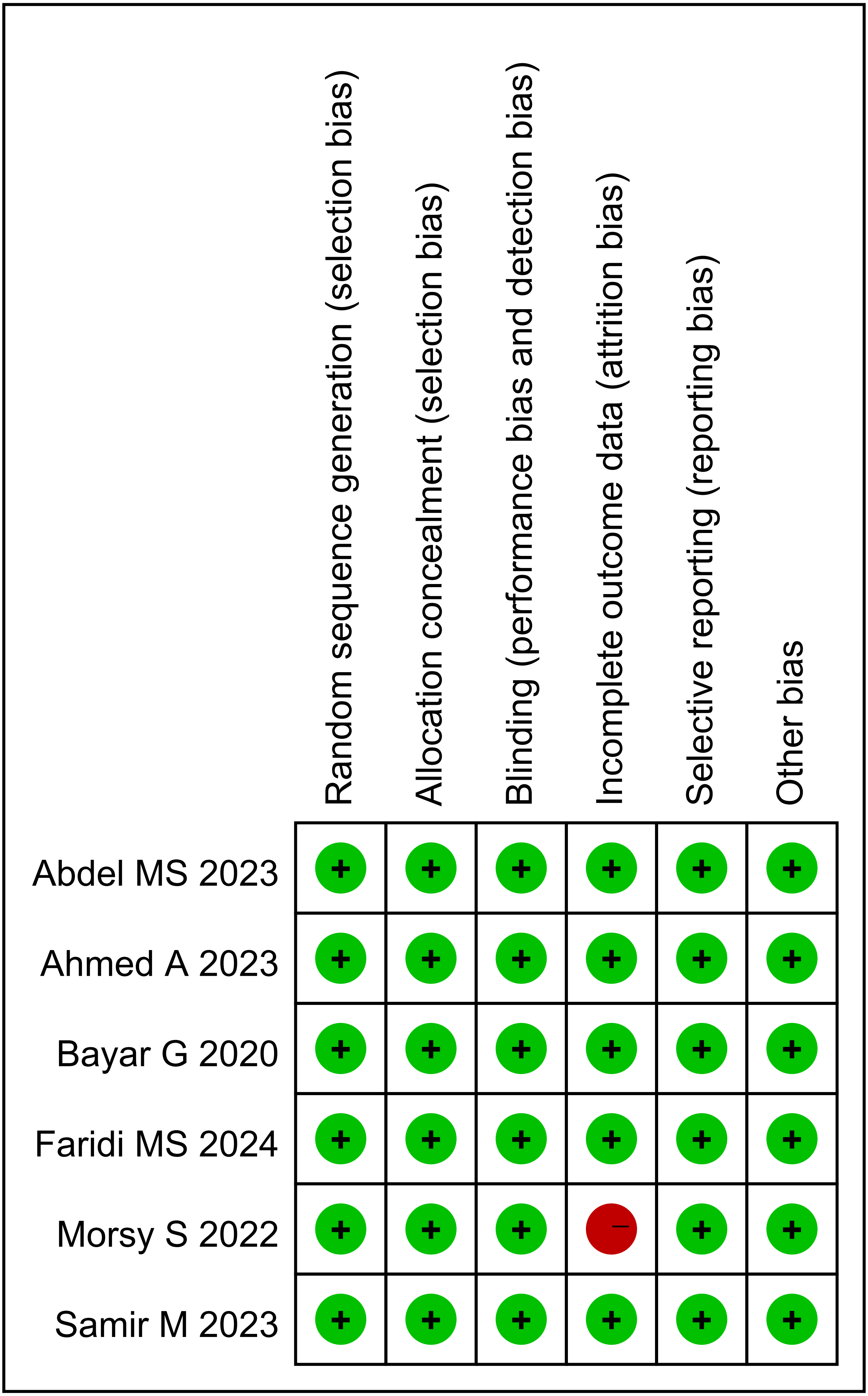
Random sequence generation (selection bias)
Allocation concealment (selection bias)
Blinding (performance bias and detection bias)
Incomplete outcome data (attrition bias)
Selective reporting (reporting bias)
Other bias
Abdel MS 2023
Ahmed A 2023
Bayar G 2020
Faridi MS 2024
Morsy S 2022
Samir M 2023
+
–
