# Supplementary figures and images for: Evaluating the safety and effectiveness of α-blockers versus mirabegron for medical expulsive therapy in ureteral calculi: A Systematic review and meta-analysis
Source: PLoS One. 2024 Dec 27;19(12):e0315328. doi: 10.1371/journal.pone.0315328 (PMC11676830; doi:10.1371/journal.pone.0315328)

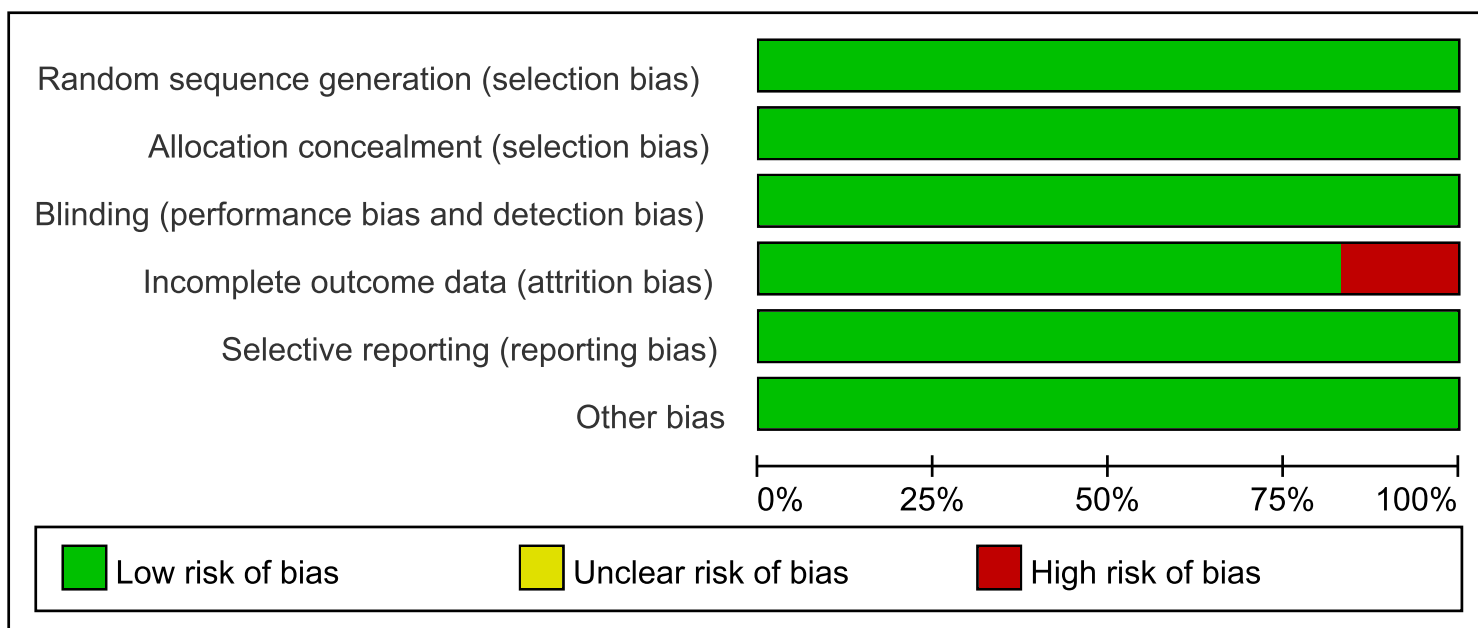

Supplement: S1 File — (ZIP) [file pone.0315328.s002.zip › Supporting information including the data extraction word file, the quality assessment figure, evaluation article/Risk of bias graph.pdf]
